# Ambient Room Temperatures in a Burn Intensive Care Unit—A Quality Improvement Project

**DOI:** 10.1177/22925503221078689

**Published:** 2022-02-11

**Authors:** Alan Rogers, George Ho, Adam Mosa, Robert Cartotto

**Affiliations:** 1 Ross Tilley Burn Centre, Sunnybrook Health Sciences Centre, Toronto, ON, Canada; 2 Department of Surgery, Division of Plastic, Reconstructive & Aesthetic Surgery, University of Toronto, Toronto, ON, Canada

**Keywords:** ambient temperature, room temperature, hypothermia, burns, hypermetabolism, burn unit, quality improvement

## Abstract

**Introduction:** Patients with major burn injuries are particularly susceptible to hypothermia. The ability to maintain and rapidly increase ambient temperatures may reduce the impact of hypothermia and the hypermetabolic response. The purpose of this study was to determine ambient patient room temperatures in a burn intensive care unit (ICU) and to evaluate our ability to adjust these temperatures. **Methods:** The ambient temperatures of 9 burn ICU patient rooms were recorded hourly over a 6-month period in an American Burn Association-verified burn centre. Temperatures were recorded using wall-mounted smart sensors, transmitted to a mobile smartphone application via Bluetooth, and then exported to Excel for analysis. On 2 predetermined dates, thermostats in all rooms were simultaneously set to maximum, and monitored over 3 h. This represented a sound change initiative, and replicated a medical order to increase the ambient temperature during critical stages of patient care. **Results:** We recorded 4394 individual hourly temperature measurements for each of the 9 rooms. The mean ambient temperature was 23.5 ± 0.3 °C (range 22.8-24). After intervention 1, ambient temperatures increased <2 °C in 7 rooms and by only 2 °C-3 °C in the other 2 rooms. The overall mean increase in temperature over 3 h across all rooms was 1.03 °C ± 1.19 °C (range −0.88 to 3.26). Following intervention 2, temperatures could be increased by ≥2 °C in only 2 rooms with an overall mean increase in temperature of only 0.76 °C ± 0.99 °C (range −0.29 to 2.43) across all rooms. **Conclusions:** The burn ICU rooms were relatively cool and our ability locally to adjust ambient temperatures quickly was limited. Burn centres should have regular facility assessments to assess whether ambient temperatures can be adjusted expeditiously when required.

## Résumé

**Introduction :** Les patients ayant des brûlures importantes (>20% de la surface corporelle totale) sont particulièrement exposés au risque d’hypothermie. La capacité à maintenir et à rapidement augmenter la température ambiante peut réduire la répercussion négative de l’hypothermie et de la réponse hypermétabolique. L’objectif de cette étude était de déterminer la température ambiante des chambres de patients dans une unité de soins intensifs (USI) pour brûlés et d’évaluer notre capacité à ajuster ces températures. **Méthodes :** La température ambiante de neuf chambres de patients en USI pour brûlés a été enregistrée heure par heure pendant une période de 6 mois dans un centre pour brûlés vérifié par l’ABA. Les températures ont été consignées en utilisant des capteurs intelligents montés sur les murs avec transmission par Bluetooth à une application mobile pour téléphone intelligent, puis exportées dans un tableau Excel pour analyse. À deux dates prédéterminées, les thermostats de toutes les chambres ont été simultanément réglés au maximum et contrôlés pendant 3 heures. Cela représentait une initiative de changement réfléchie, répliquant une consigne médicale d’augmentation de la température ambiante pendant les phases critiques de soins aux patients. **Résultats :** Nous avons enregistré 4394 relevés horaires de la température pour chacune des neuf chambres. La température ambiante moyenne était de 23,5 ± 0,3 °C (écart : 22,8 à 24). Après la première intervention, les températures ambiantes ont augmenté de moins de 2 °C dans 7 chambres et de seulement 2 °C à 3 °C dans les deux autres chambres. L’augmentation moyenne globale pendant les 3 heures dans toutes les chambres était de 1,03 °C ± 1,19 °C (écart : −0,88 à 3,26). Après la deuxième intervention, les températures ont pu être augmentées de ≥ 2 °C dans seulement deux chambres avec une augmentation globale moyenne de la température de seulement 0,76 °C ± 0,99 °C (écart : −0,29 à 2,43) pour l’ensemble des chambres. **Conclusions :** Les chambres de l’USI pour brûlés étaient relativement fraîches et notre capacité à ajuster rapidement les températures ambiantes était limitée. Les centres pour brûlés devraient faire l’objet d’évaluations régulières d’établissement pour déterminer si la température ambiante peut être ajustée dans des délais très brefs en cas de besoin.

## Introduction

Following major burn injuries, patients become profoundly hypermetabolic, and are at risk of hypothermia due to heat loss via convection, radiation, and evaporation, due to disruption of the epidermal barrier.^1,2^ While burn size is the primary determinant of the severity of the hypermetabolic response, cooling of the ambient temperature increases the metabolic rate, an effect maximally observed at an ambient temperature of 22 °C.^3^ Conversely, in patients with massive burns (>60% total body surface area [TBSA]), resting energy expenditure (REE) is reduced by increasing the environmental temperature from 25 °C to 33 °C.^4^ Patients with major burns also have an altered thermoregulatory set-point and are more comfortable in a warmer environment, typically around 30 °C.^5^

Perioperative hypothermia, defined as a core temperature below 36 °C, is accompanied by a range of ill effects in surgical patients in general, including increased risk of myocardial infarction,^6^ surgical site infection,^7,8^ and coagulopathies.^9–11^ It is assumed that these problems occur in the setting of major burn surgery where exposure, prolonged operative time, blood loss, and transfusion of fluid and blood can all contribute. A retrospective study has shown increased rates of both infectious and noninfectious complications related to intraoperative hypothermia in the context of burns.^12^

Consequently, we currently believe that it is important to maintain a warm ambient environment in the burn patient's intensive care unit (ICU) room during day-to-day care, during dressing changes, and in the immediate pre- and postoperative periods. The National Institute for Health and Care Excellence guidelines recommend that ambient temperatures in emergency departments, trauma centres, operating theatres, and ICUs should never be <21 °C,^13^ but this is probably too low for a burn centre. A recent survey found that the most commonly employed strategy for maintaining patient normothermia in North American burn centres was ambient temperature modification,^14^ but we are unaware of any studies with documented temperatures. Furthermore, we suspected that the ambient temperatures in the burn ICU rooms in our centre are considerably lower than the range of 25 °C to 32 °C that may be preferable.

Root cause analysis in a multidisciplinary forum, involving specialist burn nurses, surgeons, intensivists, and therapists, suggested that room temperatures were variable and not effectively adjusted either for patient comfort or to optimize burn care. The purpose of this quality improvement (QI) project was to accurately determine the ambient temperatures in patient ICU rooms over a 6-month period and to critically evaluate our ability to intervene and adjust these temperatures expeditiously when required.

## Methods

The ambient temperatures of 9 single-patient ICU rooms at an American Burn Association-verified regional burn centre were measured over 6 months from June 15, 2019, to December 15, 2019. Our burn unit is a 14-bed self-contained facility with 10 ICU beds and 4 step-down (ward) beds. We were able to measure temperatures in 9 of our 10 ICU rooms as one of the temperature probes was displaced during the course of the study. To answer our primary research question and test our hypothesis, ambient temperatures were measured in each room using a small, wall-mounted, smart sensor (SensorPush™) placed at standardized positions away from windows and electronic equipment and 2 m from the floor. The smart sensors collected data at 1-min intervals, had a storage capacity of up to 20 days, and could also transfer data with mean values per 15, 30, or 60 min. The sensors were automatically synchronized via Bluetooth to an encrypted mobile smartphone using the SensorPush™ application. All devices were calibrated simultaneously prior to the start of the study, as per instructions provided by the manufacturer, with temperatures and humidity across all devices standardized to ±0.2 °C and ±2%, respectively. The data were recorded continuously and simultaneously in all 9 ICU rooms from June 15, 2019, to December 15, 2019, and were exported at sample intervals of 1 h and transferred to a Microsoft Excel spreadsheet for graphing and analysis.

Two unit-wide interventions were undertaken during a 6-month period, on August 15, 2019, and September 19, 2019. On these dates, all thermostats in the 9 patient rooms were simultaneously adjusted to “maximum” and the effect was monitored over 3 h. This was identified as a sound change initiative and replicated a medical order to preemptively increase the ambient temperature during critical phases of patient care. These might include a potentially hypothermic patient returning from the operating room after a lengthy surgical procedure, or from the resuscitation area after an acute burn admission. This allowed us to evaluate the facility's capacity to increase ambient temperature expeditiously when required.

In addition to the outcome measures during the baseline and intervention stages, principal changes in ambient temperature, process, and balancing measures were also collected as per “model for improvement” methodology. These included the fidelity of the sensors to collect and store the data every hour for a 6-month period, and any reported complaints related to temperature changes in patient rooms by staff or patients themselves. The ARECCI (A pRoject Ethics Community Consensus Initiative) Ethics Screening tool^15^ indicated that the study did not require institutional research ethics board review. Demonstrating a relationship between specific patient outcomes and ambient temperatures was not the purpose of this study.

Continuous variables were reported as the mean ± standard deviation (SD). Categorical variables were described as percentages. Statistical analysis included one-way analysis of variance, which was performed using Microsoft Excel and IBM SPSS for Macintosh (Version 23.0; IBM Corp). Statistical significance was established at *P* < .05.

## Results

**Figure 1.**
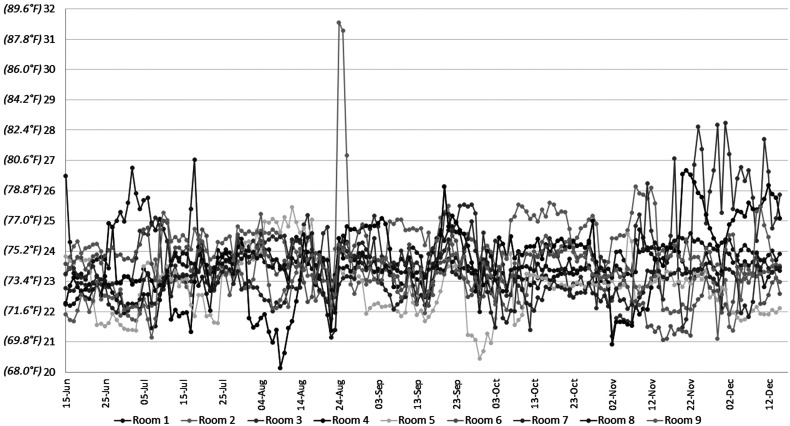
Daily mean temperatures—June 15 to December 15 (°C).

Temperature measurements were obtained every hour over a 6-month period in 9 patient ICU rooms in the burn unit (n = 9), resulting in 4394 hourly readings per room (Figure 1). Humidities and temperatures were evaluated and compared for each room and the unit average, between months and seasons of the year, but no statistically significant conclusions could be detected.

**Figure 2.**
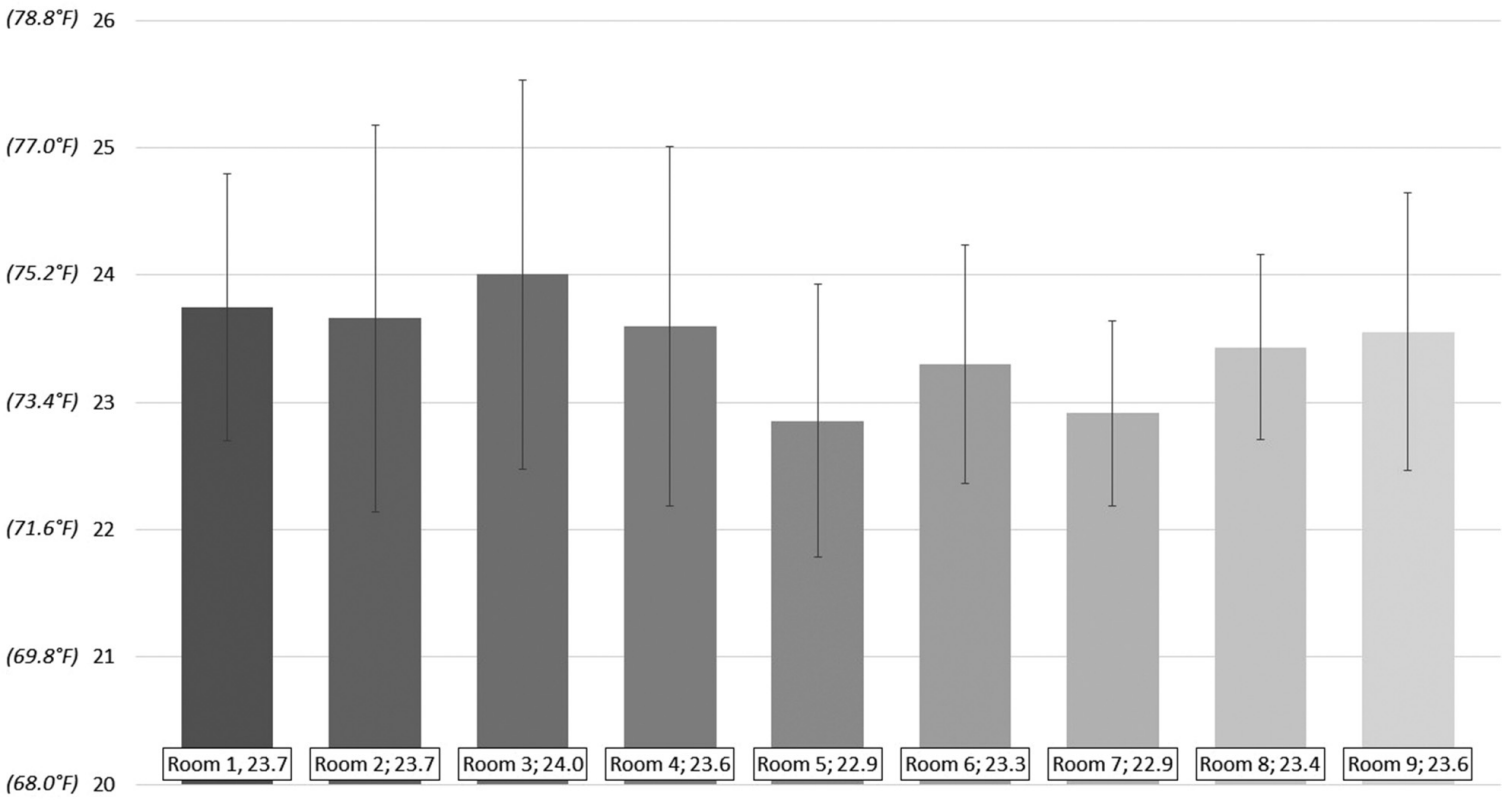
Overall mean temperatures – June 15 to December 15 (°C).

The mean ambient room temperature was 23.5 °C ± 0.3 °C (range 22.9 °C-24 °C) and the maximum and minimum recorded temperatures were 32.4 °C and 19.2 °C, respectively (Figures 1 and 2). Although the mean temperatures were similar for many of the rooms (Table 1), room 3 (24.0 °C ± 1.0 °C) was significantly warmer than room 5 (22.9 °C ± 1.1 °C) (*P* = .04). Ambient temperatures deviated below a mean temperature of 22 °C during a mean of 422 h per room (9.6% of the recorded time [range 128-1069 h; 2.9%-24% of the recorded time]), and below 21 °C during a mean of 55 h per room (1.2% of the recorded time, range 2-145 h; 0.04%-3.3% of the recorded time) (Table 2).

**Table 1.** Overall Average Monthly Room Temperatures (°C), per Room (±SD).

	Room 1	Room 2	Room 3	Room 4	Room 5	Room 6	Room 7	Room 8	Room 9
June 15 to July 14	24.51 ± 1.05	23.04 ± 0.86	23.53 ± 0.71	22.96 ± 0.65	22.71 ± 0.95	23.91 ± 0.45	22.67 ± 0.42	22.58 ± 0.57	22.68 ± 1.10
July 15 to August 14	23.96 ± 0.78	23.63 ± 0.68	23.92 ± 0.49	22.52 ± 1.19	23.64 ± 1.20	23.72 ± 0.45	22.88 ± 0.40	23.53 ± 0.42	23.90 ± 0.88
August 15 to September 14	23.50 ± 0.77	24.20 ± 0.93	23.89 ± 0.90	23.86 ± 0.67	22.87 ± 0.90	23.50 ± 0.67	23.08 ± 0.77	23.39 ± 0.35	23.26 ± 0.54
September 15 to October 14	23.50 ± 0.73	23.70 ± 0.95	23.89 ± 1.13	23.92 ± 0.87	22.39 ± 1.06	23.23 ± 0.62	23.03 ± 0.95	23.38 ± 0.32	23.75 ± 1.02
October 15 to November 14	23.55 ± 0.41	24.65 ± 0.78	23.24 ± 1.16	23.19 ± 1.15	22.99 ± 0.42	23.09 ± 0.93	22.66 ± 0.34	23.53 ± 0.40	23.80 ± 0.95
November 15 to December 15	23.51 ± 0.38	22.70 ± 0.72	25.58 ± 1.85	25.16 ± 0.90	22.50 ± 0.57	22.35 ± 0.77	23.16 ± 0.46	24.13 ± 0.24	23.87 ± 0.65

**Table 2.** Total Number of Hours Below 21 °C and 22 °C from June 15 to December 15, 2019, per Room (out of 4394 Total Hourly Readings per Room).

	Room 1	Room 2	Room 3	Room 4	Room 5	Room 6	Room 7	Room 8	Room 9
Number of hours <21 °C	50	63	29	145	108	25	2	31	39
Number of hours <22 °C	128	386	295	595	1069	402	385	194	344

**Figure 3.**
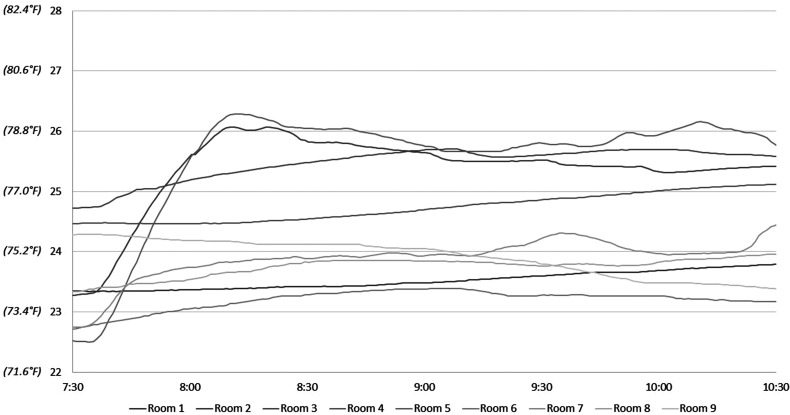
Temperatures following intervention 1—August 15 (°C).

After the first intervention on August 15, 2019, ambient temperatures increased minimally in 7 of the 9 rooms and only by 2 °C to 3 °C in the other 2 rooms (Figure 3 and Table 3). The mean temperatures before and after the first intervention were 23.49 °C ± 0.81 °C and 24.52 °C ± 0.99 °C, respectively, equating to a mean increase in 1.03 °C ± 1.19 (range −0.88 °C to 3.26 °C).

**Table 3.** Hourly Effect of Interventions 1 and 2, per Room.

	Room 1	Room 2	Room 3	Room 4	Room 5	Room 6	Room 7	Room 8	Room 9	Mean ± SD
Intervention 1										
7.30 AM (°C)	23.35	23.27	24.72	24.46	22.53	22.70	22.75	23.33	24.27	23.486 ± 0.81
10.30 AM (°C)	23.80	25.42	25.58	25.12	25.79	23.17	24.43	23.96	23.39	24.516 ± 0.99
Temp change (°C)	0.45	2.14	0.86	0.65	3.26	0.47	1.68	0.62	−0.88	1.0296 ± 1.19
Intervention 2										
11.00 AM (°C)	24.42	24.33	23.47	23.37	22.55	23.44	23.64	23.52	23.62	23.596 ± 0.55
2.00 PM (°C)	24.64	26.70	24.30	25.80	23.09	23.96	23.79	23.57	23.34	24.354 ± 1.20
Temp change (°C)	0.26	2.37	0.83	2.43	0.54	0.51	0.15	0.054	−0.29	0.7579 ± 0.99

**Figure 4.**
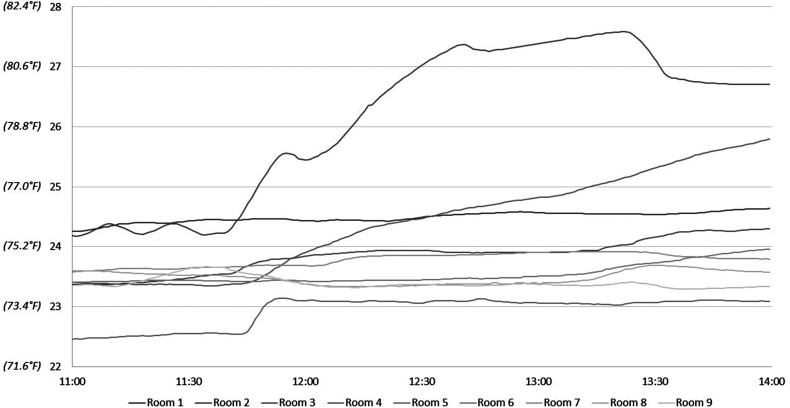
Temperatures following intervention 2—September 19 (°C).

On September 19, 2019, the mean ambient temperatures before and after the second intervention were 23.60 °C ± 0.55 °C and 24.36 °C ± 0.99 °C, respectively (Figure 4 and Table 3). The mean increase was 0.76 °C ± 0.99 °C (range −0.29 °C to 2.43 °C). Again, only 2 of the rooms could be adjusted effectively, and only by ∼2 °C.

## Discussion

This QI initiative provides insight into the ambient temperatures of burn ICU rooms in an ABA verified burn centre. We found that the average ambient temperature over a 6-month period in 9 of our burn ICU rooms was between 23 °C and 24 °C and that temperatures were below 22°C during 10% of the period studied. Furthermore, attempts to increase the ambient room temperatures within a 3-h period were unsuccessful in 7 of the 9 rooms, and when successful (in 2 of the rooms), temperatures rose by only 2 °C to 3 °C.

Baseline temperatures were colder on average than desirable burn centre target ambient temperatures, based on a survey of 52 North American burn units by Pruskowski et al,^14^ which suggested that target ambient temperatures ought to be between 24 °C and 35 °C. Whether these temperatures were actually maintained in the target range was not evaluated, and capacity to monitor and set temperatures digitally was also not captured in that survey. For many older hospitals, analog systems with “minimum–maximum” sliding scales would not allow this level of control. Our results demonstrate that when actually measured, ambient burn ICU room temperatures were below the range of intended temperature based on survey data.

The relationship between ambient temperature and amplification of the hypermetabolic response is complex. Early studies found that increasing the ambient temperature from 25 °C to 33 °C reduced the resting metabolic rate only when the burn size was at least 60% of the TBSA.^4^ Furthermore, Wilmore demonstrated that patients with major burn injuries reset their thermoregulatory set-point upwards, preferring an ambient temperature for comfort at ∼30 °C, compared to unburned controls who set an ambient temperature ∼2 °C lower for comfort.^5^ Subsequently, Kelemen et al^3^ demonstrated that at environmental temperatures between 22 °C and 28 °C, the REE rose with increasing burn size, but between 32 ° and 35 °C, mean REE (MREE) was independent of burn size, suggesting that a burn size dependent increase in MREE is promoted at cooler ambient temperatures, and developed maximally at 22 °C. This occurred across a wide range of burn sizes ≥20% TBSA. Based on these observations, our burn ICU room temperatures are a cause for concern with respect to hypermetabolism. While we did not measure REE in this study and patients with a wide range of burn sizes were treated in these rooms, we would anticipate that a mean room temperature of 23 °C to 24 °C, with temperatures <22 °C occurring 10% of the time, could have had a detrimental effect on controlling the hypermetabolic response in some patients.

In keeping with most burn centres, we make use of several measures to maintain core temperatures in addition to ambient temperature adjustments, including external heating devices and warmed intravenous fluids.^14^ There is definitive evidence to correlate perioperative hypothermia with deleterious outcomes in nonburn patients.^6–11^ In a large retrospective study by Ziolkowski et al,^12^ intraoperative hypothermia (defined as a core temperature ≤35 °C) predisposed patients to both infectious complications (relative risk [RR] 1.3, 95% confidence interval [CI]: 1.1-1.5) as well as noninfectious complications (RR 1.7, 95% CI: 1.2-1.5). Hence, the importance of a warm ambient burn ICU environment in the hours prior to burn surgery cannot be understated; it is essential that patients are not hypothermic prior to arrival in the operating room. A QI intervention study in patients with burn injuries demonstrated that simple preoperative warming with a forced air heating blanket reduced the incidence of inadvertent intraoperative hypothermia.^16^ It can therefore be extrapolated that a cool preoperative environment in the burn ICU would have the opposite effect. A review of the strategies employed to maintain normothermia in the operating room and critical care environments, failed to yield evidence for interventions in the burn population, concluding that practice is often derived from evidence in patients with markedly different perioperative physiology.^2^ While several studies in that review described the need for a warm ambient environment, it is not clear if or how this might be achieved. It may be true that the inability to tightly control room temperatures in many burn centres has precluded the conduct of meaningful prospective studies in this area.

This study highlighted our inability to quickly adjust ambient temperatures according to clinical indication, such as when a potentially hypothermic patient is imminently returning from the operating room or being transferred after trauma assessment and resuscitation with a major burn injury. These findings generated impetus to undertake broader participation in this QI initiative, including our hospital's Power Plant and Operations Maintenance Department. A central facility thermostat is responsible for the burn centre's room temperatures and manual thermostats should have the ability to adjust individual room temperature up or down by 5°C. As a result of this study, there are plans to replace the current temperature control systems in line with our need for more accurate regulation and adjustment when required. They are also undertaking a review of air exchanges per hour in each room, as well as the pressure gradients between the general areas and burn ICU rooms. There is limited evidence in the burn literature related to the optimal guidelines for burn unit design, but the ability to control the temperature within a certain range appears critical to optimal patient care. Similarly, infection control is improved in operating rooms and critical care areas with more than 20 air exchanges per hour, but this might come at the expense of an optimal ambient temperature. Gus et al^17^ recently highlighted that the patient ICU room is preferably best positive pressure, with a negative pressure anteroom with filtration and expulsion systems, and a neutral common nursing area. Many older burn units would need a major renovation to be in line with this model.

Despite general agreement that higher ambient temperatures promote patient normothermia, warm conditions may be detrimental to the comfort of staff and even impair performance.^18^ Increased temperature was found to mildly impair ICU staff performance in 94.2% of surveyed burn units and 36.5% believed there was a potential for negative influence on patient care.^14^ Some propose that staff perspiration in a clean or sterile operative field may increase wound infections, or that warm conditions predispose to bacterial proliferation on the patient's wounds, in their lines, tubes, and on room surfaces. In the arthroplasty literature, sweating surgeons were found to generate significantly more bacterial colony-forming units on surgical drapes compared to nonsweating surgeons.^19^ Half of the burn directors reported the belief that perspiration may contaminate the sterile field during central line insertion.^14^

We acknowledge the limitations of this study. We sought to accurately determine the ambient temperatures and assess our ability to adjust these if required. We did not measure REE and did not establish if the cool temperatures increased REE. Similarly, we did not correlate ambient temperatures with outcomes such as infection, transfusion requirements, weight loss, length of stay, or survival. Patient-reported outcomes measuring comfort in awake patients with burn injuries, at various temperature and humidity settings, would also be interesting and of value. We did not receive any concerns from patients or staff during the 2 interventions, which is in keeping with the fact that temperatures could not be raised sufficiently for the temperature change to even be noticed. One important positive balancing measure was that over the course of 6 months of temperature monitoring, we were able to objectively demonstrate 3 low and 2 very high temperatures in patient rooms arising from nursing staff and patient observations: 3 of these were related to a fault in our facilities that could be rapidly addressed by our maintenance service. This study does not advocate that patient rooms should be maintained at a high temperature at all times. Instead, it motivates discussion about the importance of ambient temperature control and research to inform practice.

Burn centres should have regular routine facility engineering assessments to ensure that ambient burn ICU room temperatures are maintained within the range desired by the treating clinicians and their patients, and can be adjusted effectively. Further QI initiatives include setting the smart sensors to actively alert staff when room temperatures fall below a designated threshold of 22 °C.

In conclusion, hypothermia and hypermetabolism are associated with a range of deleterious consequences for patients with burn injuries. Few studies have formally documented temperatures in burn ICU rooms; the rooms evaluated in this study were relatively cool, and our ability to increase ambient temperatures quickly was limited. Burn centres should promote a culture of awareness of the risks associated with hypothermia and obtain regular room temperature measurements and facility engineering assessments.

## Highlights

- The mean ambient temperature in patient rooms in this burn ICU was 23.5 °C.
- Attempts to rapidly increase ambient temperatures were relatively ineffective and response varied markedly between rooms.
- All burn centres should undertake regular facility engineering assessments.
- Future QI initiatives are required to enable burn ICU staff to identify, monitor, and adjust the ambient room temperatures.
